# Class II PI3Ks at the Intersection between Signal Transduction and Membrane Trafficking

**DOI:** 10.3390/biom9030104

**Published:** 2019-03-15

**Authors:** Jean Piero Margaria, Edoardo Ratto, Luca Gozzelino, Huayi Li, Emilio Hirsch

**Affiliations:** Department of Molecular Biotechnology and Health Sciences, Molecular Biotechnology Center, University of Turin, 10126 Turin, Italy; jmargari@unito.it (J.P.M.); e.ratto@dkfz-heidelberg.de (E.R.); luca.gozzelino@unito.it (L.G.); huayi.li@unito.it (H.L.)

**Keywords:** PIK3C2A, PIK3C2B, PIK3C2G, PI3K-C2α, PI3K-C2β, PI3K-C2γ, PI3K68D, *piki-1*, membrane trafficking, signal transduction

## Abstract

Phosphorylation of inositol phospholipids by the family of phosphoinositide 3-kinases (PI3Ks) is crucial in controlling membrane lipid composition and regulating a wide range of intracellular processes, which include signal transduction and vesicular trafficking. In spite of the extensive knowledge on class I PI3Ks, recent advances in the study of the three class II PI3Ks (PIK3C2A, PIK3C2B and PIK3C2G) reveal their distinct and non-overlapping cellular roles and localizations. By finely tuning membrane lipid composition in time and space among different cellular compartments, this class of enzymes controls many cellular processes, such as proliferation, survival and migration. This review focuses on the recent developments regarding the coordination of membrane trafficking and intracellular signaling of class II PI3Ks through the confined phosphorylation of inositol phospholipids.

## 1. Introduction

Phosphoinositide 3-kinases (PI3Ks) are a family of enzymes involved in the phosphorylation of the 3′ position of the inositol group. The family is divided into three classes based on their structure and substrate specificity [1]. While class I and III have been widely studied [2,3,4,5,6], class II had been poorly explored until its emerging functions were discovered in the past few years.

**Figure 1:**
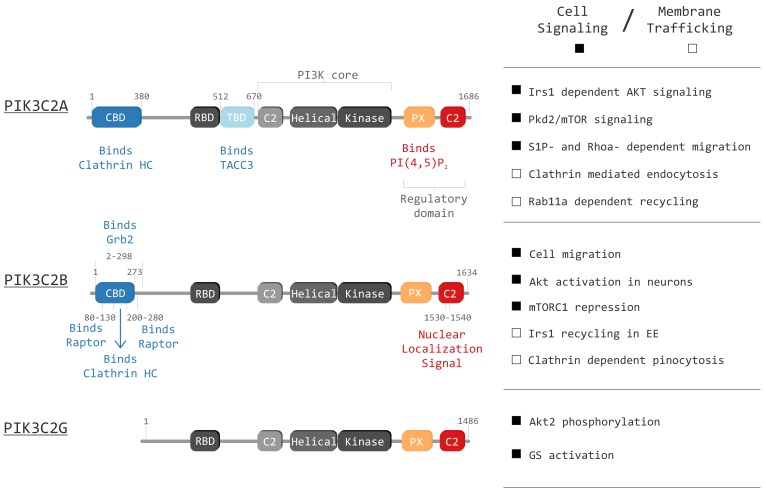
Graphical representation of class II phosphoinositide 3-kinases (PI3Ks) in mammals (PIK3C2A, PIK3C2B and PIK3C2G) and their domains: Clathrin binding domain (CBD), Ras binding domain (RBD), TACC3 binding domain (TBD), C2 membrane interacting domain (C2), Helical domain (Helical), Kinase domain (Kinase) and Phox homology domain (PX). The right panel displays known functions of each isoform. The right panel highlight principal cell signaling (black squares) and membrane trafficking (white squares) roles of the three isoforms.

The class II PI3Ks are conserved in worms, flies, mice and humans [7,8,9,10,11] (HomoloGene:20581). The first class II PI3K gene described was *Pi3K68D* in Drosophila, also represented by the ortholog *piki-1* in worm. In mammals, class II PI3Ks are composed by 3 paralogs *PIK3C2A*, *PIK3C2B* and *PIK3C2G* characterized by a common set of domains (Figure 1) [1]. This class of PI3Ks is absent in yeast, suggesting this new class of enzymes was acquired in metazoans to control complex cellular processes required in tissue development and cell to cell communications.

**Figure 2:**
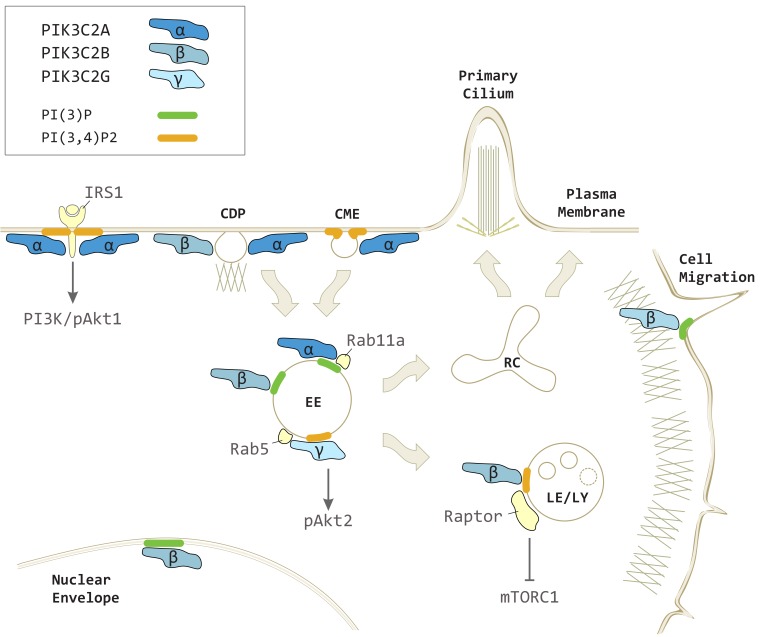
PIK3C2A, PIK3C2B and PIK3C2G in vesicular trafficking and intracellular signaling. PIK3C2A produces localized pools of PI(3,4)P2 on plasma membrane contributing to clathrin-mediated endocytosis (CME) and insulin receptor substrate 1 (IRS1) mediated class I PI3K-dependent phospo-Akt1 (pAkt1) signaling. PIK3C2A generates a pool of PI(3)P on early endosomes (EE) promoting recycling processes toward the recycling compartment (RC) and primary cilium. PIK3C2A and PIK3C2B both participate to clathrin dependent pinocytosis (CDP) on plasma membrane. PIK3C2B produces PI(3,4)P2 on late endosomes/lysosomes (LE/LY) to repress mTORC1 signaling through Raptor. PIK3C2B generates a localized pool of PI(3)P on EE during insulin signaling, on nuclear envelope, and at the leading edge during cell migration. PIK3C2G is recruited on EE by Rab5 to produce PI(3,4)P2 and increases phospho-Akt2 (pAkt2) levels.

Similarly to class I PI3Ks, class II PI3Ks are able to produce under specific conditions three different phospholipids in vitro [12]. Interestingly, while class I produces PI(3,4,5)P3 and PI(3,4)P2, and class III generates PI(3)P in vivo [1,13], class II has overlapping but distinct selectivity with the capability to produce both PI(3)P and PI(3,4)P2 in vivo [14,15,16,17,18,19]. Class II PI3Ks display a strong resistance to pharmacological inhibition by pan PI3K inhibitors like wortmannin [10,11]. Also, in the absence of the class II PI3K crystal structure, a few low potency class II PI3Ks selective inhibitors are present nowadays [20,21], despite recent studies unveiling the involvement of these enzymes in biochemical and cellular functions. Class II PI3Ks are expressed in several tissues and produce distinct phosphoinositides on spatially defined membrane sections under different conditions (Figure 2) [9,10,22,23]. In particular, *Pik3c2a* and *Pik3c2b* are expressed in a wide range of tissues where they are catalytically active in several sub-cellular compartments [15,16,18,24,25,26]. On the contrary, *Pik3c2g* is expressed in a restricted number of tissues, and have been associated with the production of a single phosphoinositide product until now [19].

## 2. Class II PI3Ks Lipid Products

Class II PI3Ks are characterized by the production of two lipid products PI(3)P and PI(3,4)P2, originating from phosphorylation on the 3′OH of the precursors PI and PI(4)P, respectively. While there is evidence showing the in vitro and in vivo products of these 3 isoforms, the precise cellular localization and timing of substrate generation and signal transduction is still debated. Growing evidence suggests that these two mechanisms are closely linked, often showing phospholipids that recruit membrane trafficking factors, thereby influencing receptor localization and signal transduction.

### 2.1. Class II Derived PI3P

The main product of PIK3C2A and PIK3C2B enzymatic activity is PI(3)P, both in vivo and in vitro [27], with PI being the preferential substrate [10,22]. PIK3C2-derived PI(3)P pools are observed in different districts of the cell, suggesting specific functions of the isoforms depending on the cellular process in which they are involved (Figure 1, right panel) (Figure 2).

With regards to overlapping functions, Pik3c2a and Pik3c2b induced PI(3)P pools are involved in cell signaling by contributing to insulin stimulation response [18,28], cell migration [29,30] and growth factor receptor response [17,31]. Further to the above consideration, the only cellular compartment in which all class II isoforms have been observed is the early endosome, where both Pik3c2a and Pik3c2b generate apparently distinct pools of PI(3)P regulating transferrin receptor and insulin receptor trafficking, respectively [18,32].

Besides these overlapping functions and localizations, Pik3c2a has a unique role in producing a PI(3)P pool responsible for mammalian target of rapamycin (mTOR) signaling [24,33] and primary cilium biogenesis [14]. However, regarding this latter role, a recent paper describes a compensatory effect of PIK3C2B in human patients lacking PIK3C2A, which could be explained by a contribution of the beta isoform in the organelle formation [34]. With respect to Pik3c2b, unique PI(3)P pools are produced at the nuclear envelope allowing cell cycle progression [35], and at the plasma membrane controlling immune cells K+ channel activity [36].

### 2.2. Class II Derived PI(3,4)P2

Besides PI(3)P, all three isoforms of class II PI3Ks are able to produce PI(3,4)P2 in vivo and in vitro [10,15,16,19,22,37]. However, while PI(3)P can be synthetized on the same membranous structure by different isoforms, the production of PI(3,4)P2 appears to be more isoform-specific depending on the compartment (Figure 2).

At the plasma membrane, PIK3C2A is involved in clathrin mediated endocytosis, where it produces a restricted pool of PI(3,4)P2 during clathrin coated pit (CCP) formation [16]. The recruitment of the PIK3C2A to the plasma membrane is mediated by its clathrin binding domain [12], thereby increasing the possibility of interaction between this kinase and its substrate PI(4)P, which is abundant on the plasma membrane, for the formation of the PI(3,4)P2.

The PI(3,4)P2 generated by Pik3c2a is also important for the phosphorylation of Akt1 during insulin receptor substrate 1 (Irs1) signaling on the plasma membrane, thus underlying a double role of the lipid substrate as a membrane trafficking and intracellular signaling player [38]. Interestingly, it has been demonstrated that the major role of the PI(3,4)P2 produced by PIK3C2G is to sustain the insulin signaling from the internal membranes [19]. Indeed, after ligand binding and activation of the insulin receptor on the plasma membrane, the receptor is internalized into the endosomal compartment, from which it is either recycled to the plasma membrane or digested in the lysosome. After insulin stimulation, PIK3C2G promotes the formation of a specific pool of PI(3,4)P2 spatially localized on early endosomes, which is necessary for long term-Akt2 activation, thus propagating and sustaining the insulin signaling [19]. Therefore, it is possible that PIK3C2A and PIK3C2G cooperate to initiate and sustain insulin signaling, respectively, at least in the tissues where PIK3C2G is expressed.

Part of the early endosome material is conveyed to late endosomes and lysosomes. A recent study proposed that PIK3C2B is responsible for the production of PI(3,4)P2 on late endosomal membranes [15]. Marat et al. showed that under conditions of serum deprivation, PIK3C2B is able to interact with Raptor on lysosomes and late endosomes, generating a pool of PI(3,4)P2 that inhibits mTORC1 activity. The beta isoform thus also displays a close link between membrane trafficking and signaling mechanism.

## 3. PIK3C2A

### 3.1. PIK3C2A Structure

*PIK3C2A*, the most well characterized PI3K class II member, is ubiquitously expressed in human cells. Structurally, besides the PI3K core domain (C2 domain, helical domain and bilobed kinase domain), PIK3C2A also harbors an additional C2 domain, a Phox homology (PX) domain located at the C-terminal region, a Ras-binding domain (RBD), a Clathrin HC binding domain (CBD) and a recently characterized TACC3 binding domain (TBD).

Although PIK3C2A shares the Ras-binding domain with class I PI3Ks, its significance for the interaction with small GTPases is still unknown. Besides its kinase domain and Ras-binding domain, PIK3C2A holds an unstructured N-terminal region showing high sequence similarity with the clathrin interactor protein AP-3β3A and evidences a clathrin interaction region. Indeed, Gaidarov et al. showed that the first 144 amino acids of PIK3C2A N-terminal region are able to directly interact with clathrin. Furthermore, they revealed that localized PI(3)P production by PIK3C2A in clathrin coated pits affects the clathrin mediated endocytosis and sorting in the trans-Golgi network [12]. Accordingly, additional analysis using tandem mass spectrometry also identified that Pik3c2a is present in the isolated rat brain clathrin coated vesicles, which further validates the involvement of Pik3c2a in clathrin mediated trafficking [39].

Recently, emerging evidence has suggested that PIK3C2A enzymatic activity plays an essential role in primary cilium formation [14], vesicle trafficking [16,32], and cell migration [30]. Modulation of PIK3C2A activation has been reported to depend on several receptors including tyrosine kinase receptors, such as insulin receptor (IR) [40], epidermal growth factor receptor (EGFR) [41], transforming growth factor beta receptor 1 (TGFBR1) [26], vascular endothelial growth factor receptor (VEGFR) [17], as well as G-protein coupled receptors such as sphingosine-1-phosphate receptor 1 (S1PR1) [30], and C-X-C motif chemokine receptor 2 (CXCR2) [42]. The conserved kinase domain present in all the PI3Ks confers to PIK3C2A the ability to generate three different phosphoinositides in vitro: PI(3)P, PI(3,4)P2 and PI(3,4,5)P3. However, the results of in vitro assay showed that the activity of PIK3C2A towards PI(4,5)P2, the substrate for class I PI3Ks, was less than 1% of the total and it was detectable only in the presence of phosphatidylserine [22]. In line with this finding, besides the PI(3)P production, Gaidarov et al. reported an increased in vitro activity towards PI(4)P and PI(4,5)P2 when clathrin binds PIK3C2A N-terminal inhibitory domain (1–142).

At the C-terminal region, PIK3C2A presents a PX-domain that has been structurally resolved. Similarly to its N-terminal region, this domain is involved in the targeting of the protein to the plasma membrane by binding to specific phosphoinositides [43]. A recent study with structural analysis by hydrogen/deuterium exchange mass spectrometry (HDX-MS) revealed that the PX-C2 domains folds back onto the kinase domain, causing an auto-regulation on its basal activity. Destabilization of this intramolecular contact increases PIK3C2A activity and leads to accumulation of its lipid product, and increased endocytosis [44]. Furthermore, recent research on PX and C2 domains of PIK3C2A found that C2 domain is able to bind the phosphoinositide enriched membrane but interestingly this process can be blocked by elevated calcium concentration [45].

### 3.2. PIK3C2A in Vesicular Trafficking and Signaling

#### 3.2.1. Endocytosis through Dynamin-Dependent and -Independent Mechanisms

PIK3C2A mainly generates PI(3,4)P2 and PI(3)P, which are highly enriched at the plasma membrane and in early endosomes [46]. The reduction of *PIK3C2A* expression decreases the amount of PI(3,4)P2 and causes mislocalization of endocytic route markers, like the transferrin receptor [12]. Mechanistically, PI(3,4)P2 synthesis was described as an essential mediator of the late stages of clathrin-mediated endocytosis (CME). Accordingly, changes in PIK3C2A localization may represent key events required for membrane trafficking. Indeed, results by time-lapse microscopy show that PIK3C2A is recruited to CCP immediately after their formation, on which it produces a PI(3,4)P2 pool necessary for SNX9 binding to the neck of the forming vesicle. This process promotes the recruitment of Dynamin, a GTPase involved in the scission of newly formed vesicles, and facilitate the maturation of CCP into clathrin coated vesicles (CCV). Thus, when *PIK3C2A* is silenced by RNA interference, a drastic decrease in the levels of PI(3,4)P2 is accompanied by a delay in CCP maturation. Intriguingly, the overexpression of an exogenous *PIK3C2A* mutant only producing PI(3)P is incapable of restoring the CCP maturation defect in cells. This indicates that the regulation of PI(3,4)P2 by PIK3C2A is essential for the clathrin dissociation and maturation of CCV into early endosomes [16]. Moreover, a recent study highlighted the role of PIK3C2A in the control of another receptor internalization process named clathrin dependent pinocytosis (CDP), however, whether the kinase activity of this isoform is involved is still not clear [47].

Furthermore, recent research has found the involvement of Pik3c2a activity in dynamin-independent endocytosis in which small GTPases, such as Rhoa, Cdc42 and Arf6, regulate membrane invagination, elongation and vesicle scission. Interestingly, the activity of Rhoa has been found to be regulated by Pik3c2a [48]; it is thus tempting to speculate an involvement of Pik3c2a in dynamin-independent endocytosis via small GTPase regulation. Krag et al. found that the internalization of several proteins is mediated by PIK3C2A through dynamin-independent pathways. Downregulation of *PIK3C2A* prevented internalization of CD59 by impairing the recruitment of early endosome antigen 1 (EEA1) to vesicular compartments, which indicates a general role for PIK3C2A in regulating physiologically relevant dynamin-independent internalization pathways [49].

#### 3.2.2. Recycling and Exocytosis

During endocytosis, PIK3C2A produces PI(3,4)P2, however its main lipid product in vitro is PI(3)P [10], which regulates the maturation, sorting and motility of endosomes [50].

In muscle cells, Pik3c2a is activated by insulin stimulation and translocates to the plasma membrane [28]. Subsequently, activated Pik3c2a promotes the internalization of glucose receptor, Slc2a4, by the production of PI(3)P; however, the generated PI(3)P on the endocytic compartment is phosphorylated into PI(3,5)P2 by Pikfyve, and in turn favors the translocation of protein complexes to the plasma membrane [28,33]. In human endothelial cells, PIK3C2A plays an important role in vessel formation and integrity contributing to the early embryonic lethality and impaired tumor angiogenesis in the *Pik3c2a* mutant mice. Yoshioka et al. found that PIK3C2A promotes Rhoa, Rac1 and Rap1 activation upon VEGF and S1PR1 mechanical stimulation. The silencing of *PIK3C2A* causes a decrease in the number of PI(3)P enriched endosomes, impaired endosomal trafficking, defective delivery of vascular endothelial cadherin to endothelial cell junctions and defective junction assembly [17]. In addition, similar results have also been found in Drosophila melanogaster. The single class II PI3K in Drosophila melanogaster Pi3K68D, produces PI(3)P in the endosomal compartment. PI(3)P is then converted to PI by the myotubularin Mtm (Drosophila MTM1) PI3-phosphatase found in a complex with Sbf (Drosophila MTMR13 pseudophosphatase, also a Rab21 GEF). Surprisingly, this sequence of events is necessarily required in endosomes exocytosis toward the plasma membrane. During this process, several effectors are indispensable; Rab21 in particular is thought to interact not only with PI(3)P but also within a Pi3K68D/Sbf/Mtm complex, which favors its activation [51]. Similarly, in mice, Pik3c2a is localized in the pericentriolar recycling endocytic compartment at the base of primary cilium, where it regulates the production of a PI(3)P pool necessary for the activation of Rab11a. In mouse embryonic fibroblasts, silenced for *Pik3c2a*, Rab11a activation and the accumulation of Rab8 on the primary cilium are reduced, causing a defect in Smo ciliary translocation and Sonic Hedgehog (Shh) signaling, ultimately impairing embryonic development [14]. As a critical member of the Rab GTPase super family, Rab11a plays an important role in the microtubule regulated transport via myosin, kinesin and dynein interactions, which are required for membranous cargos trafficking [52]. Rab11a controls trafficking processes from sorting endosomes to the endosomal recycling compartment by binding the cytoplasmic dynein subunits 1 and 2 (DYNC1LI1 and DYNC1LI2) via the Rab11a effector protein FIP3 [53,54]. Recently, we revealed that PIK3C2A derived production of PI(3)P on early endosome evokes the Rab11a activation which, in turn, specifically recruits the phosphatidylinositol 3-phosphatase myotubularin 1 (MTM1), but not other MTM family members, and helps in the release of cargo from early endosome [32]. Accordingly, this transport is mediated by dynein motor proteins, which directs the recycling cargo towards recycling endocytic compartment. Consistently, previous works on PI3Ks in Drosophila melanogaster and Caenorhabditis elegans showed that MTM1 antagonizes the class II- and class III-derived PI(3)P pools on endosomal membranes [55,56,57,58]. In neuronal cells, Pik3c2a derived PI(3,4)P2 is an important signaling molecule during early development and is critical in regulating actin aggregation and neuritogenesis [59]. Moreover, PIK3C2A also participates in the process of G protein–coupled receptors (GPCRs) delivery from trans-Golgi network in neurons. In particular, Daniel et al. show that PIK3C2A regulates the surface delivery of the delta opioid receptor (δR) and δR-mediated cyclic adenosine monophosphate (cAMP) inhibition. Further, they display how this mechanism is not only present in neuronal cells, but also in epithelial cells [60,61].

#### 3.2.3. Scaffolding Effect on Mitotic Spindle Assembly

Recently, several studies showed the involvement of PIK3C2A in the regulation of carcinogenesis and metastasis [62,63]. Gulluni et al. reported a direct and causal relationship between breast tumor growth and Pik3c2a, in which Pik3c2a can bind clathrin and TACC3 complex as a scaffold for stabilizing microtubules and mediates spindle stability during mitosis. Pik3c2a scaffold function confers a drug resistance to taxanes, anti-microtubule agents, in neoadjuvant treatment during chemotherapy. Furthermore, breast cancer development in NeuT mice which are heterozygous for *Pik3c2a* has a biphasic propagation with an initial delay in tumor onset followed by the emergence of fast-growing clones. This is the first tumor suppressor function identified in the family of the PI3Ks [64]. While there is clear evidence that PIK3C2A scaffold function acts as a tumor suppressor, there is a lack of knowledge concerning its kinase function in cancer progression. Given the role of PI(3)P in Rab11a activation, and the evidence showing the effect of Rab11a and its effector Rab11fip3 in cancer cell motility [65], we cannot exclude an hidden oncogenic potential in PIK3C2A kinase activity.

In conclusion, PIK3C2A is important for endocytosis and cargos recycling by controlling the generation of phosphoinositides including PI(3)P and PI(3,4)P2.

## 4. PIK3C2B

### 4.1. PIK3C2B Structure

*PIK3C2B* is ubiquitously expressed in human tissues. Structurally, it shares with the other class II PI3Ks a well conserved PI3K core domain, composed by a central C2 domain, a helical domain and a kinase domain. Moreover, additional C2 and phosphoinositide-binding PX domains are present at the C-terminal of the protein. Indeed, it is reported that PIK3C2B can associate with phospholipids, such as lysophosphatidic acid (LPA), mediating its recruitment to the plasma membrane. Given the high similarity of PIK3C2A and PIK3C2B lipid binding domains, it is possible that PIK3C2B could interact with PI(4,5)P2, which is known to activate PIK3C2A at the plasma membrane. It is reported that, in the cytoplasm, the PX-C2 module folds back onto the kinase domain of PIK3C2A inhibiting its enzymatic activity, but is essential for the complete activation on PI(4,5)P2-positive membranes [44]. Since loss of PIK3C2B C-terminal C2 domain enhances the in vitro activity of the enzyme [22], we cannot exclude that this regulatory mechanism is shared between PIK3C2A and PIK3C2B.

The C-terminal C2 domain can also localize PIK3C2B at the nuclear membrane. It is reported that a small fraction of PIK3C2B is present in the nucleus in quiescent cells, but translocation from the cytosol to the nucleus is greatly increased in the presence of epidermal growth factor (EGF), together with an overall increase of the enzymatic activity in the nucleus [31]. Translocation to the nucleus was also observed in studies on PIK3C2A, in which the authors identified a nuclear localization signal formed by a cluster of basic amino acids at the terminal C2 domain [66]. Given the high similarity of the domain between PIK3C2A and PIK3C2B, it is possible that the KRKTKxxxK sequence could also act as a localization signal in PIK3C2B.

The large N-terminal region is thought to exert a regulatory function on the activity of PIK3C2B. The N-terminal mediates the interaction with EGFR and the scaffold protein Grb2 after EGF stimulation, increasing the activity of PIK3C2B [67]. Notably, deletion of proline rich sequence in this region greatly enhances the kinase activity of the protein [68]. The N-terminal region also contains a clathrin binding domain and two newly discovered regions that are able to interact with Raptor in the mTOR complex 1 [15].

Finally, PIK3C2B structure also includes a Ras-binding domain, predicted on the homology with the class I PI3Ks RBD. However, to date there is no evidence of a direct interaction between Ras and PIK3C2B.

### 4.2. PIK3C2B Signaling in Vesicular Trafficking

PIK3C2B is a key controller of endosomal trafficking. It is reported that PI(3)P is important for the conversion of very early adaptor protein phosphotyrosine interacting with PH domain and leucine zipper 1 (APPL1)-positive endosomes into early endosome antigen 1 (EEA1)-positive endosomes [69] and its production might be in part controlled by Pik3c2b, at least in metabolically active tissues [18]. In accordance, impairment of the catalytic activity of PIK3C2B resulted in an increased number of APPL1-positive endosomes in hepatocytes compared to wild type (WT) cells. APPL1-positive vesicles were more dispersed in the cytoplasm, while EEA1-positive early endosomes and Rab7-positive late endosomes were found to be irregularly shaped and enlarged. However, loss of PIK3C2B activity did not affect the starvation-induced autophagic vesicles, indicating that PIK3C2B selectively acts on endosomal trafficking. In this scenario, PIK3C2B inactivation resulted in an alteration of the insulin receptor trafficking and a class I PI3K-dependent AKT serine/threonine kinase (AKT) activation. The proposed mechanism is in accordance with other works that show how EGF-stimulated Pik3c2b activity in neurons significantly results in Akt activation [70].

PIK3C2B was also recently found to localize on lysosomes and late endosomes in nutrient deprivation conditions, where it inhibits mTORC1 signaling [15]. Upon serum withdrawal, PIK3C2B is recruited by Raptor and produces a pool of PI(3,4)P2. However, the mechanisms by which PIK3C2B is recruited and how the mTORC1 signaling is shut down remains largely unclear.

A recent study also highlighted the role of PIK3C2B in clathrin dependent pinocytosis (CDP) [47]. Single knockdown of *PIK3C2B*, without affecting *PIK3C2A* expression, resulted in complete inhibition of the CDP, indicating that the enzymes play non-redundant roles. It has been proposed that PIK3C2A is recruited to the clathrin-coated structures through its binding to PI(4,5)P2 on the membrane, while PIK3C2B interacts with the scaffold protein Intersectin 1 (ITSN1) through its proline-rich domain [47]. ITSN1 can recruit proteins for the actin rearrangement of the pinocytic process, such as FCH And Double SH3 Domains (FCHSD2). Since FCHSD2 localization also needs the presence of PI(3,4)P2 signaling, PIK3C2B may contribute to actin polymerization by generating PI(3,4)P2 and recruiting FCHSD2.

Different studies linked PIK3C2B to cancer cell migration and lamellipodia/filopodia formation [29,71,72]. In particular, in prostate cancer, PIK3C2B was able to promote cell invasion in a MEK/ERK independent mechanism, promoting the expression of the transcription factor Slug, a well-known epithelial-mesenchymal transition (EMT) inducer [73]. In ovarian cancer, knockdown of *PIK3C2B* significantly inhibited the formation of lamellipodia and reduced the number of metastasis in human cancer cell xenografts [74]. Moreover, the study showed that treatment with ceramide liposomes, which are believed to have multiple activities against the progression of ovarian cancer, reduced cell migration by affecting PIK3C2B compartmentalization. In fact, Kitatani et al. demonstrated that ceramide interacted with PIK3C2B and led to its relocalization away from lamellipodia, inhibiting cell motility and metastasis.

A recent study highlighted the role of Pik3c2a and Pik3c2b in uterine smooth muscle contraction [75]. Single knockouts did not have any effect on muscle contractile ability, thus suggesting a compensatory mechanism between the two isoforms. On the other hand, double knockouts for *Pik3c2a and Pik3c2b* impaired uterine contraction and normal parturition. No change in calcium channels expression or activity were observed, but the Rho-Rho kinase pathway was diminished. However, the molecular mechanisms linking class II PI3Ks and Rho still need to be clarified.

## 5. PIK3C2G

### 5.1. PIK3C2G Structure

Several lines of evidence suggest that PIK3C2A and PIK3C2B are linked to endosomal trafficking, but very little is known about the regulation and function of the third class II member, PIK3C2G.

Recent studies increased our knowledge about the functional role of this isoform. In particular, PIK3C2G, in contrast to other class II members which are broadly expressed, is predominantly present in exocrine glands like liver and pancreas but also in breast, prostate and small intestine [19,76]. Pik3c2g expression is increased during liver regeneration after partial hepatectomy in a time-dependent manner, underlining a possible role in some yet-undefined liver-specific matured functions [37]. Besides its different pattern of expression, PIK3C2G displays different domain organization compared to the other class members. The main difference is that it doesn’t possess the clathrin binding domain, unlike PIK3C2A and PIK3C2B, suggesting its inability to induce clathrin-mediated endocytosis. Moreover, PIK3C2G lacks also the highly basic consensus sequence KRKTKxxxK, which has been demonstrated to be important for the nuclear localization of PIK3C2A [66].

### 5.2. PIK3C2G in Metabolic Signaling and Vesicular Trafficking

In the absence of *Pik3c2g*, the endosomal pool of PI(3,4)P2 is severely reduced and the major impact is a reduction of the delayed and sustained insulin-dependent Akt2 phosphorylation. The lack of *Pik3c2g* seems to have a specific effect on Glycogen synthase (GS) activation; while no other alteration in Akt effectors was found (like the mTORC1/Rps6kb1 axis and the Foxo1-3 transcription factors) [19]. As observed in *Pik3c2g* deficient mice, the reduction in GS activity induced a decrease in hepatic glycogen storage and increased circulating glucose levels, which is sufficient to induce age-related insulin resistance. Moreover, compensatory processes occurred, such as a boost in triglycerides production, which finally led to hyperlipidemia, an increase in fat mass and fatty liver particularly in response to a high-fat diet, thus underlying an important role for Pik3c2g in controlling cell metabolism by acting at the vesicular level. Rab5, an important player in the endosomal system, is orchestrating this mechanism. Indeed, insulin induces the colocalization of Pik3c2g with Rab5-positive early endosomes. Moreover, it has been demonstrated that Rab5 can induce the translocation of Pik3c2g on EE by directly binding it. The active form of Rab5, Rab5-GTP, can also recruit the adaptor protein Appl1, which promotes the preferential association/activation with the specific Akt2 isoform. Accordingly, Rab5 has double role in controlling the delayed and sustained insulin-dependent Akt2 activation on EE, through the recruitment of Pik3c2g and Appl1 [19].

Results obtained using *Pik3c2g* null mouse model are supported by different genome wide association studies (GWAS). The first study links *PIK3C2G* single nucleotide polymorphism (SNPs) with an increased risk of developing type 2 diabetes in a Japanese population [77]. This result was strengthened by another study, in which the authors found a correlation between SNPs in *PIK3C2G* and diabetic nephropathy in three different GWAS databases [78]. Moreover, another study reported an association between *PIK3C2G* polymorphisms with hyperlipidemia and myocardial infarction [79]. Finally, a recent study associated SNPs in *PIK3C2G* with high body mass index [80]. All of this evidence highlights an important function of *PIK3C2G* in regulating cell metabolism.

Beyond metabolic disorder, PIK3C2G has also been found altered in different types of cancer. Mutations in the *PIK3C2G* gene were significantly associated with poor prognosis in patients with intrahepatic cholangiocellular carcinoma (ICC) [81]. A recent study observed that stage III colorectal cancer patients treated with oxaliplatin with low copy number of *PIK3C2G* had a 2.44-fold increased probability of recurrence and 2.51-fold increased risk of death [82].

In conclusion, not only could *PIK3C2G* be an important biomarker in metabolic disorders like diabetes, due to its role in controlling cell metabolism at early endosome, it could also be used as a biomarker in specific types of cancer for recurrence and survival.

## 6. Concluding Remarks

Class II PI3Ks are emerging as important enzymes in regulating membrane trafficking routes and their related signaling cascades. Increasing evidence supports the non-redundant functional role acquired by the three isoforms in mammals. These complementary functions are mainly achieved by the production of the PI(3)P and PI(3,4)P2 lipid substrate localized in different membranous compartments.

Excluding the differential expression in tissues and during development, all the class II PI3Ks, including their single non-mammalian ortholog *piki-1* and *Pi3K68D*, are involved in endolysosomal functions, with a tight link to small GTPases Rabs and Rhoa, as well as nutrient sensing complexes such as mTORC1 in many cell types. The investigation of the close interplay between these enzymes will allow to untangle the complex interaction between the various class II PI3Ks products and their role in integrating cell signaling and membrane trafficking in the endolysosomal system.

We can conclude that beside the vast amount of knowledge acquired in the last several years on class II PI3Ks, there are still several mechanisms that need to be understood and elucidated to improve our knowledge of this class of enzymes in basic research and physiological contexts.
